# Resource Estimations in Contingency Planning for Foot-and-Mouth Disease

**DOI:** 10.3389/fvets.2017.00064

**Published:** 2017-05-11

**Authors:** Anette Boklund, Sten Mortensen, Maren H. Johansen, Tariq Halasa

**Affiliations:** ^1^Veterinary Institute, Technical University of Denmark, Copenhagen, Denmark; ^2^The Danish Veterinary and Food Administration, Head Office, Glostrup, Denmark; ^3^Veterinary Control Office North, Herning, Denmark

**Keywords:** stochastic modeling, veterinary crisis, epidemics, simulation models, preparedness

## Abstract

Preparedness planning for a veterinary crisis is important to be fast and effective in the eradication of disease. For countries with a large export of animals and animal products, each extra day in an epidemic will cost millions of Euros due to the closure of export markets. This is important for the Danish husbandry industry, especially the swine industry, which had an export of €4.4 billion in 2012. The purposes of this project were to (1) develop an iterative tool with the aim of estimating the resources needed during an outbreak of foot-and-mouth disease (FMD) in Denmark, (2) identify areas, which can delay the control of the disease. The tool developed should easily be updated, when knowledge is gained from other veterinary crises or during an outbreak of FMD. The stochastic simulation model DTU-DADS was used to simulate spread of FMD in Denmark. For each task occurring during an epidemic of FMD, the time and personnel needed per herd was estimated by a working group with expertise in contingency and crisis management. By combining this information, an iterative model was created to calculate the needed personnel on a daily basis during the epidemic. The needed personnel was predicted to peak within the first week with a requirement of approximately 123 (65–175) veterinarians, 33 (23–64) technicians, and 36 (26–49) administrative staff on day 2, while the personnel needed in the Danish Emergency Management Agency (responsible for the hygiene barrier and initial cleaning and disinfection of the farm) was predicted to be 174 (58–464), mostly recruits. The time needed for surveillance visits was predicted to be the most influential factor in the calculations. Based on results from a stochastic simulation model, it was possible to create an iterative model to estimate the requirements for personnel during an FMD outbreak in Denmark. The model can easily be adjusted, when new information on resources appears from management of other crisis or from new model runs.

## Introduction

Foot-and-mouth disease (FMD) is a highly contagious disease, which is known to spread easily within and between herds and cause severe economic losses in each herd as well as in the country (1). The control and eradication of FMD within the EU is governed by EU legislation (Council Directive 2003/85/EC of 29 September 2003; http://eur-lex.europa.eu/legal-content/EN/TXT/PDF/?uri=CELEX:32003L0085&from=EN). Following the directive, EU member states are obliged to use a stamping out policy, involving quarantine, movement restrictions, zoning, and slaughter and disposal of all affected herds, followed by cleaning and disinfection (CD) of the farm. Additional control measures can be used, for example, preemptive culling or vaccination, after approval of the plan by the European Commission regulatory committee *Standing Committee on the Food Chain and Animal Health*.

Preparedness planning for a veterinary crisis, such as an FMD outbreak, is important in order to be fast and effective in the eradication of the disease. For countries with a large export of animals and animal products, each extra day in an epidemic will cost millions of Euros due to the closure of export markets. This is of utmost importance for the Danish swine industry, which had a yearly export of €4.4 billion in 2012 (2).

Modeling results have previously been used to inform decision-making related to disease control options (3–14). Models have traditionally been including limitations on resources for culling and vaccination, while only few models have included resources for other things such as surveillance visits [e.g., Ref. (15)]. With this work, we propose to also use the outputs of simulation modeling for planning and operational purposes in a Veterinary Administration of a country before and during a veterinary crisis.

Geering and Lubroth (16) describe how the first step in preparing a resource plan is to make a resource inventory, “listing all the resources needed to respond to a moderate-sized FMD outbreak or other high-priority emergency disease. The plan includes personnel, equipment, and other physical resources.” Garner et al. (17) estimated the needed resources during a hypothetical FMD-epidemic in Australia, focusing on the 90th percentile out of 100 iterations simulated to set-off from the area previously predicted to give the worst epidemics in terms of size and duration (18). For the Danish Veterinary and Food Administration, it is important to know how many persons must be available within hours and days in order to efficiently handle and eradicate the disease. And to consider whether these people are already available in the organization and how extra personnel can be recruited. Furthermore, it is important to consider, whether this personnel has the required level of education or if extra training and education is needed. Similarly, the need for materials and services during a veterinary crisis must be identified and quantified. These materials and services include for example cars, sampling materials and testing capacity at the laboratory, equipment for culling of animals, protective clothing, disinfection agents, valuators, trucks and rendering capacity.

The purposes of this project were (1) to develop an iterative tool with the aim of estimating the resources needed during an outbreak of FMD in Denmark and (2) to identify areas, which can be bottle necks in the veterinary administration, and thereby delay the control of the disease and the time to regain disease free status and the access to export markets. The tool developed should easily be updated, when new knowledge is gain from other veterinary crises or during an outbreak of FMD.

## Materials and Methods

### The Simulation Model

The DTU-DADS model (version 0.16) was used to simulate spread of FMD in Denmark (19, 20).

#### Farm Data

From the official Danish central herd register, all Danish herds registered with cattle, swine, sheep, or goat in the period from October 1st 2006 to September 30th 2007 were extracted and used in the model. This period was used to avoid influence of the blue tongue outbreak in Denmark, which started in October 2007. For each herd, a unique identification number, the herd type, the numbers of animals of different types, and the UTM geo-coordinates was extracted. In total, 23,550 cattle herds, 11,473 swine herds, and 15,830 sheep or goat herds were included. Sheep and goat herds were grouped in one category, called sheep, as there are a limited number of goat herds in Denmark, and they are considered to be handled similar to sheep herds during an epidemic. Furthermore, herds were described as different types, cattle herds as milking or beef cattle, sheep herds as commercial or hobby herds, and swine herds as 19 different herd types, based on their SPF status and their production type (5). For each herd, the rate of daily movements were calculated as the total numbers of movements off the herd in the 1-year period mentioned above divided by 365, for batches of animals moved to other herds or to abattoirs, respectively. For swine herds, animals moved to other herds were divided into sows or weaners. Farms including several species were separated into several herds, with different herd IDs but with the same coordinates.

#### Modeling Spread of Disease

Spread of disease was modeled to occur through seven different spread mechanisms: (1) direct contact, i.e., animal movements, (2) indirect medium risk contacts, i.e., veterinarians, artificial inseminators, or milk controllers, (3) indirect low risk contacts, i.e., visitors, feed stuff/rendering trucks, (4) abattoir trucks, (5) milk tankers, (6) markets, and (7) local spread.

Based on movement data from the period October 2006 to September 2007, the rate of movement per day for the individual herd was used as λ in a Poisson distribution simulating spread of disease. Similarly, the rate of abattoir deliveries was calculated for the individual herd and used as λ in a Poisson distribution simulating the risk of spread on the abattoir route. Contrary, the pick-up of milk from dairy herds was simulated as a Poisson distribution with λ = 0.6 for all dairy herds. Indirect medium and low risk contacts were simulated with different λ for different herd types (5, 19). Markets were simulated for cattle only, as markets in Denmark are restricted to cattle and horses, with an average of 3.5 extra contacts generated from a market. And local spread was simulated as a probability of spread within 3 km from infected herds, simulating the unexplained spread within short distance as a consequence of for example limited airborne spread, rodents, birds, flies, and animal movements or person contacts not registered (19, 20).

#### Modeling Detection of Disease

Detection of the first infected herd was simulated to occur between day 18 and 23 after the disease was introduced, with a mode of 21 days. Thereafter, the disease could be detected either from the farmer or veterinarian (basic surveillance) or from surveillance by official veterinarians as result of tracing contacts from infected herds or as surveillance in the protection or surveillance zones. The probabilities of detection by each type of surveillance were assumed to be dependent on the herd type, as different species show more or less clinical signs (19).

#### Modeling Control of Disease

After detection of disease, a set of control measures must be applied based on the EU council directive 2003/85/EC. These include depopulation of all detected herds, CD of infected properties, tracing (back and forward) of contacts, and establishment of protection (3 km) and surveillance (10 km) zones around detected herds. In both zones, movements of animals are prohibited and all herds must be surveyed at least twice in the protection zone and once in the surveillance zone, before the zones can be lifted.

In the model, herds were assumed to be depopulated as soon as capacity was available. A daily capacity for depopulation of 2,400 ruminants and 4,800 swine was estimated by the industry and the Veterinary and Food administration based on experiences with other diseases (19). Animals moved from a detected herd within 14 days before detection were assumed to be traced, and the receiving herd to be culled. For other traced contacts, the herds receiving contact were assumed to be put under surveillance. Traced contacts and herds in the protection zone would be visited as soon as possible, depending on available resources for surveillance. A daily capacity for surveillance was estimated to 450 herds by the Veterinary and Food administration, based on experiences with other diseases (19). Herds in the protection and the surveillance zone would be set for surveillance visit immediately after the initiation of the zones, and herds in the protection zone would be set for another visit after 21 days and before lifting the zone. Details on how surveillance is modeled can be obtained from Halasa and Boklund (15). All sheep within the zones were simulated to be tested as described in the Danish contingency plan due to non-specific clinical signs in sheep (21). The probability of detecting disease from clinical surveillance and testing increased with time (5).

Furthermore, a 3-day national standstill for all animal movements was modeled, based on the Danish contingency plan for FMD.

#### Initiation of Disease Spread and Model Run

One thousand cattle herds were randomly chosen and used to initiate the spread of disease (index herds). Epidemics starting in cattle herds have previously been shown to create some of the largest outbreaks under the simulated circumstances (5, 19).

For each index herd, one iteration was run, resulting in 1,000 epidemic simulations. The outcome of the model included, for every iteration and for every day in the epidemic, which herds were detected, which herds were depopulated, and which herds were surveyed. Of the 1,000 simulated epidemics, in 19 cases, the disease did not spread from the first infected herd and was not detected, resulting in 981 simulated epidemics in total.

### Estimations of Resources during an Outbreak

A working-group of 12 persons^1^ was constituted with staff from the Danish Veterinary and Food Administration (10 persons), with experience in contingency planning and handling of veterinary crises, and experts from the Danish Emergency Management Agency (DEMA) (1 person) and the National Veterinary Institute (1 person). A series of meetings were undertaken, in order for these experts to identify best practices for all work tasks during an epidemic and estimate the man power and other resources needed. In some cases, information was from external sources, while other information was exclusively based on the knowledge and experience within the group.

Estimates for resources during an outbreak were divided in resources for detected herds, suspected herds, surveyed herds, and local crisis centers (Tables 1 and 2). No assumptions were made regarding the skills required for neither different tasks nor the manpower available, except for the resource assumptions in the model, described in Section “Modeling Control of Disease.” Neither did we decide on whether veterinarians (VET) should be official veterinarians, vets from private practice or from other sources. “Technicians” (TECH) are defined as non-vets working as animal technicians or as legal advisors, HR, or IT personnel. Administrative personnel (ADM) were only related to work in the local crisis center (LCC). The DEMA is hired to be involved in the culling and cleaning phase on detected herds. They will be taking care of setting up an organizational board at the farm, cleaning and disinfecting people and trucks entering and leaving the farm, preliminary CD of the farm, and eventually transportation of culled animals. Personnel from DEMA were categorized as leading officers, officers, and recruits. For all groups of personnel, a detailed description of tasks and necessary skills was provided in the Danish report from the project (22).

**Table 1:** **Inputs used for estimation of the total personnel resources needed during a foot-and-mouth disease epidemic in Denmark (brackets refer to Eq. 1)**.

Task (t)	Description	Output from simulation model (g)	Team (team)	Estimate (k)
Detected herds				
Valuation	Valuation of animals in detected herds for compensations to the farmer	#Detected herds	3 assessor and 1 vet	A team can per day assess
				1 cattle herd or
				2 swine herds or
				4 sheep herds
				1 car per team is needed

Culling	Culling of all animals in detected herds	#Animals in depopulated herds	1 coordinating vet	Per hour
			Cattle—1 vet, 4 technicians, 1 truck driver	12 cattle
			Swine—1 vet, 8–10 technicians, 2 truck drivers	30 sows or 60 finishers or 300 weaners
			Sheep—1 vet, 1 technician	20 sheep

Cleaning and disinfection point		#Depopulated herds	1 vet in 4 h	

Clinical examination and sampling	60 animals in detected herds are assumed to be sampled	#Depopulated herds	1 vet, 1 technician	Per day: 1 herd
	All animals are clinically evaluated			

Cleaning and disinfection	Preliminary CD	#Depopulated herds	1 vet	Per day: 1 cattle or swine herd 4 sheep herds
	Personnel from the Danish Emergency Management Agency (DEMA)	#Depopulated herds	2 leading officers	2 days in each cattle/swine herd
	Personnel from the DEMA	#Depopulated herds	9 officers	2 days in each cattle/swine herd
	Personnel from the DEMA	#Depopulated herds	47 recruits	2 days in each cattle/swine herd

Final cleaning and disinfection	Conducted by subcontractors, but managed by this team	#Depopulated herds	1 vet	5 days in cattle and swine herds^a^
				0.5 day in sheep herds^a^

**Suspicions**	We have assumed to have five suspected farms for each detected herd			
Clinical suspected farms	Fence, clinical inspections, testing, epidemiological interview, tracing	5 × #detected herds	1 vet, 1 technician	Per day: 1 herd

**Surveillance in zones and in traced contact herds**	The day of the surveillance is extracted from the simulation model			
			1 vet	
	Clinical surveillance	#Surveyed herds		Per day: 4 cattle or swine herds
	Collection of blood samples	#Surveyed herds		4 sheep herds

*Vet, veterinarian*.

*^a^Divided over 21 days*.

**Table 2:** **Inputs used for estimation of the total personnel resources in local crisis centers (LCCs) during a foot-and-mouth disease epidemic in Denmark**.

Input	Description	Team	Estimate
LCCs^a^	We assume that there will be three crisis centers active at all times during the epidemic. Management, communication, and competency development is in total, not per crisis center		
Management (lead)		2 vet, 1 lawyer, 1 adm, 1 technician pr. crisis center, 1 human resource	
Log and journals (log)		Min. 3, 1 adm	Per 5 detected herds
Press and communication (press)		1 technician	Per crisis center
Case-officers (case)		1 adm	Per 3 detected herds
Suspicion group (suspicion)		1 vet, ½ adm	Per 2 SI the first 2 weeks, per 3 SI thereafter
Assessor (valuation)		1 adm	Per 2 detected herds
Culling (cull)		1 vet	Per 2 detected herds
Cleaning and disinfection		Counted as part of detected herds	
Epidemiology		1 vet, ½ adm	Per 3 detected herds in the first 2 weeks, Per 4 detected herds thereafter
Screening (screening)		1 adm	Per 50 detected herds
Movements and dispensations (MoveDisp)		1 vet, 1 adm	First week Thereafter
		8 vets, 24 adm	
Service and catering (service)		2 adm	Per 100 persons
Logistics, equipment (log)		2 technicians, 5 adm	Per 100 persons
Personnel administration (HR)		1 human resource person	All epidemic
Competency development (Edu)		1 vet, 1 adm	Per crisis center

*Vet, veterinarian; Adm, administrative personnel*.

*^a^Names in brackets refer to the abbreviations used in the R-script (Supplementary Material)*.

Based on the daily outputs from the simulation model, personnel for valuation of herds for each day (i) in the epidemic was calculated as the total type of personnel (p) needed, i.e., the numbers of VETs, TECHs, ADMs, and staff from DEMA, for a given task (t), here valuation, and a given species (a) as:

(1) $${\text{Total}}_{\text{p,a,i,t}}=\sum \text{animals}{\left(\text{or herds}\right)}_{\text{g,a,i}}\cdot {\text{team}}_{\text{p,t,a}}\cdot \frac{1}{K_{\text{t,a}}}$$

where a is reflecting the animal species, g is the action these animals are undergoing—i.e., detection, depopulation, or surveillance, team_p,t,a_ is the estimated team for a given type of personnel, task, and species, and *K*_t,a_ is the number of animals (or herds) of a given species that a team can handle per day for the given task.

For valuation, the number of veterinarians needed would then be calculated based on Eq. 1 as:

(2) $${\text{Total}}_{\text{VET,a,i,valuation}}\text{=}\sum {\text{herds}}_{\text{detection,a,i}}\cdot {\text{VET}}_{\text{valuation,}}\cdot \frac{\text{1}}{K_{\text{valuation,a}}}$$

where VET_valuation_ is the number of VETs in the valuation team and *K*_valuation_ is the number of herds a valuation team can handle in one day (Table 1).

The total number of veterinarians needed for depopulation was calculated as:

(3) TotalVET,a,i,depop=∑herdsdepop,at,i⋅coordinatingVet+∑animalsdepop,at,i⋅VETdepop⋅1workingHours⋅Kdepop,at

where at is now reflecting the animal species and type of animal—i.e., cattle, sheep/goat, sows, finishers, or weaners, VET_depop_ is the number of VETs in the depopulation team, and *K*_depop_ is the number of animals a depopulation team can handle per working hour. The numbers of coordinating vets at the herd is a constant, with 1 as the default value. Working hours was estimated from the working group to be 8 h efficient work a day, excluding transport time, and breaks.

The number of veterinarians needed for the cleaning and disinfection point (CDP) of the herd was calculated as:

(4) $${\text{Total}}_{\text{VET,a,i,CDP}}\text{=}\sum {\text{herds}}_{\text{depop,a,i}}\cdot \text{CDP}\cdot {\text{VET}}_{\text{CDP}}$$

where CDP is the numbers of CD points in a herd (default = 1), and VET_CDP_ is the number of days that a veterinarian will be needed at the CDP (default = 0.5). The CDPs were assumed to be used in cattle and swine herds only, based on the limited herd sizes of Danish sheep and goat herds.

The numbers of veterinarians used for clinical inspection (CI) in detected cattle and swine was calculated as:

(5) $${\text{Total}}_{\text{VET,a,i,CI}}=\sum {\text{herds}}_{\text{depop,a,i}}\cdot {\text{VET}}_{\text{CI}}\cdot \frac{\text{1}}{K_{\text{CI,a}}}$$

where VET_CI_ is the numbers of veterinarians in the team used for CI in the herd and *K*_CI_ is the number of herds a CI team can handle in one day. Because of the limited size of Danish sheep herds, CI and blood sampling was assumed to be included in the culling of the animals in sheep herds.

The numbers of veterinarians used for preliminary CD of detected herds was calculated as:

(6) $${\text{Total}}_{\text{VET,a,i,CD}}\text{=}\sum {\text{herds}}_{\text{depop,a,i}}\cdot {\text{VET}}_{\text{CD,a}}\cdot \frac{\text{1}}{K_{\text{CD,a}}}$$

where VET_CD_ is the numbers of veterinarians in the team used for initial CD of the herd and *K*_CD_ is the number of herds a CD team can handle in 1 day.

The following final cleaning and disinfection (FCD) of herds was assumed to be done by private commercial cleaning companies, but under guidance and acceptance by the official veterinarians. Therefore, for each species, a certain time was needed for the veterinarians, but spread over a 3 week period of time, as few hours are needed a day. This was calculated as:

(7) $${\text{Total}}_{\text{VET,a,i,FCD}}=\frac{\sum {\text{herds}}_{\text{depop,a,i}}\cdot {\text{VET}}_{\text{FCD,a}}\cdot \frac{\text{1}}{K_{\text{FCD,a}}}}{{\text{duration}}_{\text{FCD}}}$$

where duration_FCD_ is the time period over which the FCD is taking place and *K*_FCD_ is the number of herds a FCD team can handle in 1 day. This value is then included every day over the duration_FCD._

When an epidemic is running, there will be suspicion of disease, also in herds that are not infected. Suspicions (SI), which are following detected with FMD as result of investigation, will in the model be counted as detected herds. However, there will be suspicion of FMD in non-infected herds as well.

As the simulation model only simulate spread of infection, we do not have information on non-infected SI from the model. Therefore, a conservative estimate based on data from the UK 2001 epidemic was used, i.e., five SI per detected herd was assumed for the numbers of inspections based on passive surveillance (23).

The SI were randomly distributed over a period of 10 days, starting the day after a herd was detected in the model. As it is not known in which herd type a suspicion will occur, we could not take herd type into account for SI. The number of veterinarians needed to inspect SI of FMD was calculated as:

(8) $${\text{Total}}_{\text{VET,i,SI}}=\sum {\text{herds}}_{\text{suspicion,i}}\cdot {\text{VET}}_{\text{SI}}\cdot \frac{1}{K_{\text{SI,a}}}$$

where VET_SI_ is the number of veterinarians used in the team investigating a suspicion and *K*_SI_ is the number of herds a suspicion inspection team can handle in 1 day.

The numbers of veterinarians needed for surveillance in traced herds and in herds in the protection or surveillance zones (zoneSurv) were calculated as:

(9) $${\text{Total}}_{\text{VET,i,zoneSurv}}=\sum {\text{herds}}_{\text{surveillance,a,i}}\cdot {\text{VET}}_{\text{zoneSurv}}$$

where VET_zoneSurv_ was the numbers of veterinarian needed for a surveillance visits. No difference was assumed between herd types for surveillance visits. From the output of the simulation model, the day of the surveillance visit was extracted and, therefore, eventual waiting time for a surveillance visit was already accounted for.

During an epidemic, a LCC will be created according to the Danish veterinary contingency plan (24). The numbers of LCCs in Denmark could vary from 1 to 3, related to the regions for official veterinarians. It was assumed that all LCCs were active from the beginning to the end of the epidemic. The needed numbers of veterinarians were calculated as a total for all LCCs (Table 2). After the first 14 days after first detection, it was assumed that the experience in the crisis centers would result in more effectiveness in the centers and, therefore, the time needed for different work tasks would be reduced (Table 2). The numbers of veterinarians needed in the LCCs were calculated as:

(10) $$\begin{array}{cc} {\text{Total}}_{\text{VET,i,LCC}}= & \text{LCC}\cdot ({\text{LCC}}_{\text{VET,management}}+\sum {\text{herds}}_{\text{detect,a,i}}\cdot \text{suspicion}\cdot \\ \; & {\text{LCC}}_{\text{VET,suspicion}}\;+\sum {\text{herds}}_{\text{detect,a,i}}\cdot {\text{LCC}}_{\text{VET,EPI}}\\ \; & +\sum {\text{herds}}_{\text{depopulated,a,i}}\cdot {\text{LCC}}_{\text{VET,depop}}+{\text{LCC}}_{\text{VET,move}}+{\text{LCC}}_{\text{VET,comp}} \end{array}$$

where LCC is the numbers of local crisis centers, LCC_VET,management_ are veterinarians working in the management of the group, LCC_VET,suspecison_ are veterinarians working suspicisons, LCC_VET,EPI_ are veterinarians working with epidemiology of the epidemic, LCC_VET,depop_ are veterinarians working with depopulation of detected herds, LCC_VET,move_ are veterinarians working with movement restrictions in the zones, and LCC_VET,comp_ are veterinarians working with educating new staff during the epidemic.

Similarly, the needed numbers of technicians, administrative staff, and personnel from DEMA were calculated for each day and each task in the epidemic, and summed over all tasks resulting in the daily needs for personnel. Furthermore, the needs for rendering capacity was calculated for ruminants and non-ruminants, and the needed equipment for culling and testing was calculated, however, not included in this paper. Details from these calculations can be obtained from the authors.

### Materials

The simulation model as well as the calculations of resources was run using the freeware R (25). All estimated resources are presented in Tables 1 and 2 and calculations are presented above. The full model is available in the Datasheet S1 in Supplementary Material. From the stochastic simulation model, the following outputs per day of the epidemics were used as inputs in the resource calculations: numbers of detected herds, numbers of depopulated herds, numbers of animals (for each type of animal) in depopulated herds, and numbers of surveyed herds (Table 1) resulting in a stochastic model of resources needed during an outbreak. Resource estimations were calculated for every single epidemic (981) and presented as median values and 5th–95th percentiles.

### Sensitivity Analyses

The influence of estimates on the required number of staff during an outbreak was investigated by decreasing or increasing the number of vets, technicians, and administrative staff as described in Table 4. We investigated the effect of change on valuation, culling, CD, surveillance visits in herds under suspicion of disease and in herds located in protection and surveillance zones, on staff at the LCCs being more or less efficient, the influence of only 1 LCC, of DEMA present only 1 day in each herd compared to 2 days (default), and of the numbers of DEMA personnel needed. Sensitivity analyses were run in 100 iterations.

## Results

**Figure 1:**
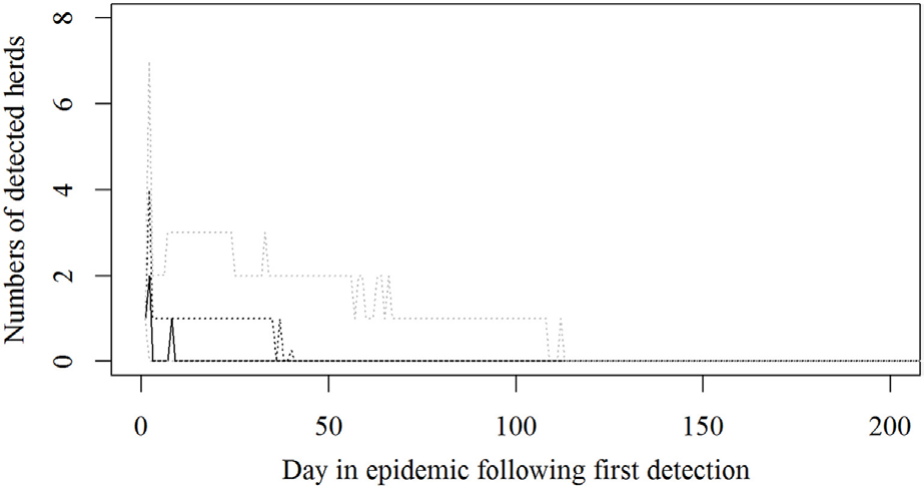
**Numbers of detected herds for each epidemic in a simulated Danish foot-and mouth disease-outbreak**. Nine hundred eighty-one iterations were simulated, all starting in a randomly selected cattle herd. The solid black line indicates the median value, the dotted black line indicates the 75th percentile, and the lower and upper dotted gray lines indicate the 5th and 95th percentiles, respectively.

The simulated epidemics had a median size of 22 (5–95%: 2–155) infected and detected herds (Figure 1) and a median duration of 34 days (5–95%: 2–142), counted from first detection until the last herd is culled, but not taking into account the time until zones are lifted. The median number of SI was 110 (5–95%: 10–7,775).

**Figure 2:**
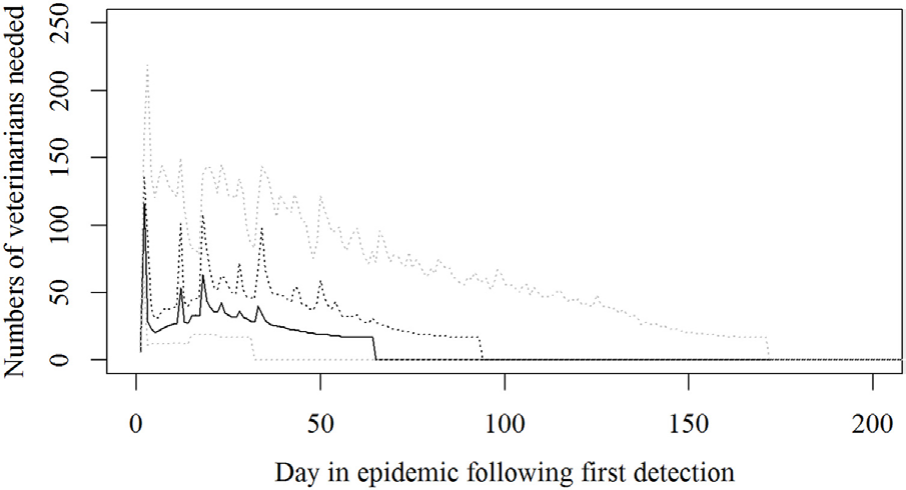
**The number of veterinarians needed during a foot-and-mouth disease (FMD) epidemic in Denmark**. Based on results from a stochastic simulation model simulating 981 FMD epidemics in Denmark, all starting in a cattle herd. The central administration is not included. The solid black line indicates the median value, the dotted black line indicates the 75th percentile, and the lower and upper dotted gray lines indicate the 5th and 95th percentiles, respectively.

**Figure 3:**
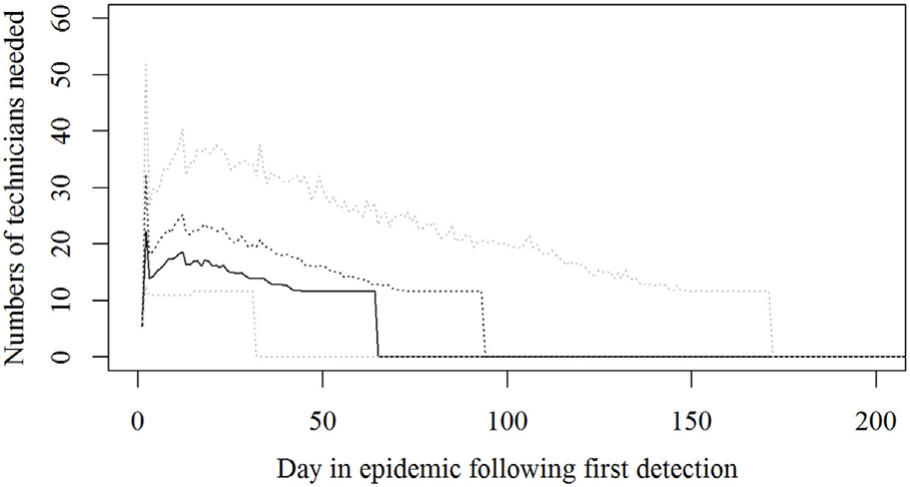
**The number of technicians needed during an foot-and-mouth disease (FMD) epidemic in Denmark**. Based on results from a stochastic simulation model simulating 981 FMD epidemics in Denmark, all starting in a cattle herd. The central administration is not included. The solid black line indicates the median value, the dotted black line indicates the 75th percentile, and the lower and upper dotted gray lines indicate the 5th and 95th percentiles, respectively.

**Figure 4:**
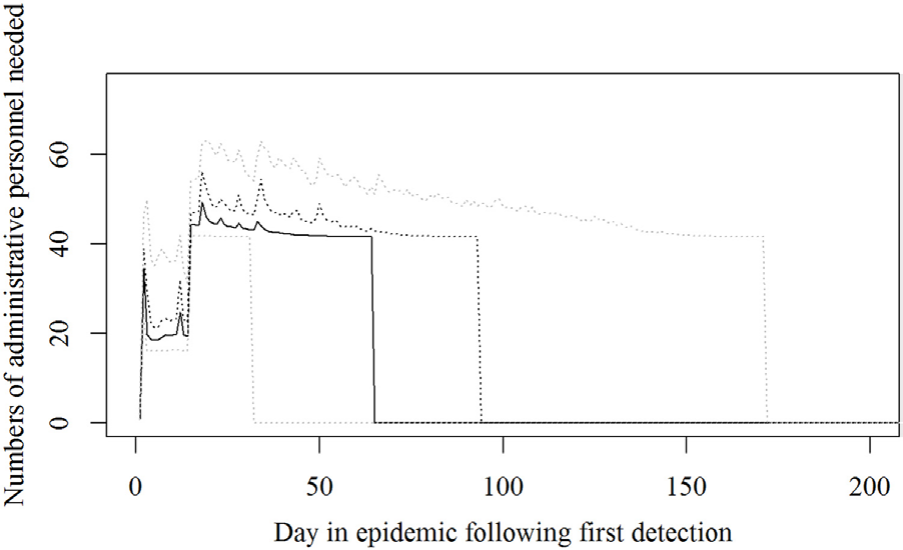
**The number of administrative personnel needed during an foot-and-mouth disease (FMD) epidemic in Denmark**. Based on results from a stochastic simulation model simulating 981 FMD epidemics in Denmark, all starting in a cattle herd. The central administration is not included. The solid black line indicates the median value, the dotted black line indicates the 75th percentile, and the lower and upper dotted gray lines indicate the 5th and 95th percentiles, respectively.

**Figure 5:**
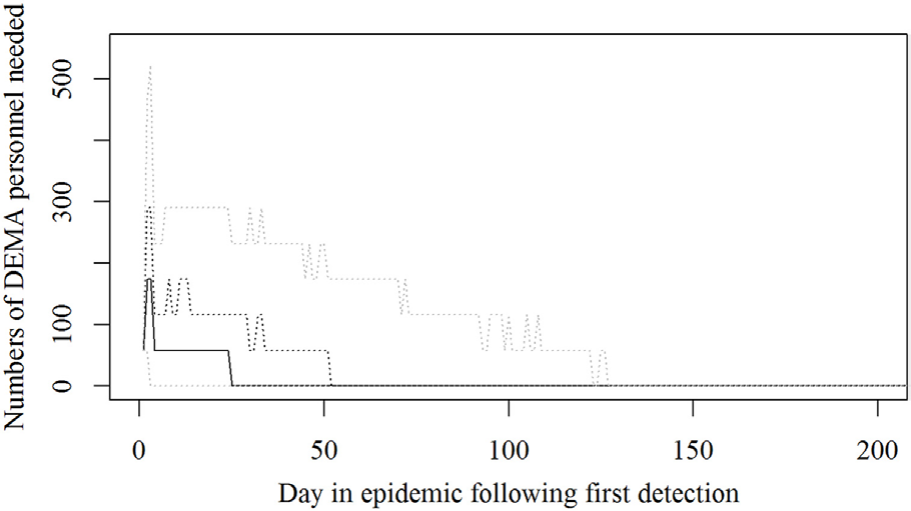
**The number of persons from the Danish Emergency Management Agency needed during an foot-and-mouth disease (FMD) epidemic in Denmark**. Based on results from a stochastic simulation model simulating 981 FMD epidemics in Denmark, all starting in a cattle herd. The solid black line indicates the median value, the dotted black line indicates the 75th percentile, and the lower and upper dotted gray lines indicate the 5th and 95th percentiles, respectively.

Based on the results from the simulation models, we estimated that the need for personnel in the regions would peak in the first couple of days with a median of 116 veterinarians, 22 technicians needed, while the need for administrative personnel would peak a little later with a need for a median of 45 administrative personnel 21 days in the epidemic (Figures 2–4; Table 3). Furthermore, the numbers of needed veterinarians would also increase at day 21, caused by the second surveillance visit of herds in the protection zone (Figure 2). Additionally, 174 persons would be needed from DEMA at day 2, mostly recruits (Figure 5; Table 3).

**Table 3:** **Estimated personnel needed at day 2, 7, 14, and 21 in 981 simulated foot-and-mouth disease-epidemics in Denmark, starting in cattle herds given as median and 5th–95th percentiles**.

	Day in epidemic			
	2	7	14	21
Veterinarians	116 (60–164)	23 (12–144)	28 (12–94)	36 (19–135)
Technicians	22 (13–52)	16(11–33)	16 (11–35)	16 (12–38)
Administrative	35 (25–46)	19 (16–39)	19 (16–31)	45 (42–61)
Danish emergency management agency	174 (58–464)	58 (0–290)	58 (0–290)	58 (0–290)

From the sensitivity analyses (Table 4), it was clear that the time needed to perform clinical surveillance in farms (either suspected farms or farms located in protection and surveillance zones) influences the estimated numbers of veterinarians and technicians needed during an outbreak. Increased efficiency in the LCCs, leading to decreased time needed for each task, decreased the need for veterinarians, technicians and administrative personnel, and using DEMA personnel for only 1 day instead of 2 in detected herds, had large influence on the total numbers of DEMA staff needed. Furthermore, the involvement of only one LCC decreased the total manpower needed during the epidemics.

**Table 4:** **Estimates of total manpower needs during a simulated Danish foot-and mouth disease-epidemic, measured as “Mann-days,” e.g., one person needed for 1 day (5th–95th percentiles)**.

Changed parameter	Change (default)	Veterinarians	Technicians	Administrative staff	Danish emergency management agency (DEMA)
Basic^a^	–	2,482 (641–9,223)	1,155 (423–3,366)	2,711 (951–7,985)	2,552 (226–20,346)
Valuation more efficient	A team can per day assess: 2 (1) cattle herd or 4 (2) swine herds or 8 (4) sheep herds	2,474 (640–9,196)	1,142 (422–3,220)	2,709 (950–7,962)	2,552 (226–20,346)
Valuation less efficient	2 (1) teams needed for each valuation	2,498 (643–9,276)	1,188 (427–3,873)	2,717 (951–8,031)	2,552 (226–20,346)
Culling working hours decreased	6 (8)	2,485 (641–9,231)	1,161 (423–3,395)	2,712 (951–7,996)	2,552 (226–20,346)
Culling working hours increased	12 (8)	2,479 (641–9,215)	1,151 (423–3,343)	2,711 (951–7,974)	2,552 (226–20,346)
Clean less efficient	Preliminary: 2 (1) vets for 1 day in cattle/swine farms	2,500 (643–9,283)	1,155 (423–3,368)	2,713 (951–7,999)	2,552 (226–20,346)
	1 vet 0.5 (0.25) day in sheep farms				
	Final: 7.5 (5) days in cattle and swine herds^b^				
	1 (0.5) day in sheep herdsb				
Clean more efficient	Preliminary: 1 vets for ½ (1) day in cattle/swine farms	2,473 (640–9,193)	1,155 (423–3,366)	2,711 (950–7,978)	2,552 (226–20,346)
	1 vet 0.125 (0.25) day in sheep farms				
	Final: 3 (5) days in cattle and swine herds^b^				
	0.25 (0.5) day in sheep herds^b^				
SuspicionVisits less efficient	2 (1) teams per day per herd	2,665 (714–9,252)	1,406 (501–4,153)	2,732 (953–7,987)	2,552 (226–20,346)
SuspicionVisits more efficient	1 team ½ (1) day per herd	2,448 (632–9,208)	1,008 (379–3,324)	2,702 (949–7,984)	2,552 (226–20,346)
SurveillanceVisits less efficient	Per day: 2 (4) herds	3,399 (784–12,796)	1,167 (426–3,443)	2,768 (961–8,234)	2,552 (226–20,346)
SurveillanceVisits more efficient	Per day: 8 (4) herds	2,121 (583–7,762)	1,149 (422–3,339)	2,688 (945–7,860)	2,552 (226–20,346)
LocalCrisisCenter, fewer	1 LCC (3)	2,072 (465–8,027)	731 (231–2,365)	2,197 (720–6,668)	2,552 (226–20,346)
LocalCrisisCenter less efficient	All personnel in LCC half as efficient	3,732 (1,071–13,100)	1,902 (749–5,438)	5,096 (1,788–14,835)	2,552 (226–20,346)
LocalCrisisCenter more efficient	All personnel in LCC twice as efficient	1,953 (427–7,629)	775 (256–2,480)	1,525 (532–4,560)	2,552 (226–20,346)
DEMA presence decreased	Days per herd: 1 (2)	2,482 (641–9,223)	1,155 (423–3,366)	2,711 (951–7,985)	1,276 (113–10,173)
Fewer DEMA staff needed	Lead Officers: 1 (2)	2,482 (641–9,223)	1,155 (423–3,366)	2,711 (951–7,985)	1,892 (168–15,084)
	Officers: 6 (9)				
	Recruits: 36 (47)				
More DEMA staff needed	Lead Officers: 3 (2)	2,482 (641–9,223)	1,155 (423–3,366)	2,711 (951–7,985)	3,256 (289–25,959)
	Officers: 12 (9)				
	Recruits: 59 (47)				

*Effect of varying parameter estimates (medians) on different resources in the resource calculations (5th–95th percentiles)*.

*^a^Sensitivity analyses are run in 100 iterations, so for comparisons, the same 100 iterations (1–100) were extracted*.

*^b^Divided over 21 days*.

## Discussion

Based on results from a stochastic simulation model, it was possible to create a model in R to estimate the requirements for personnel during an FMD outbreak in Denmark. The model can easily be adjusted, when new information on resources appear from management of other crisis, or when new simulation results are available from new model runs in peacetime. Furthermore, it is possible to adjust the model during a crisis, when model results from daily runs of the stochastic simulation model gives more precise estimates on the specific epidemic, or when adjustments in management procedures becomes available.

It was not surprising to find that especially the number of staff needed for surveillance visit influenced our results, as the numbers of herds in zones are so large. This was also in line with what was found by Garner et al. (17). This means that if veterinarians doing surveillance visits can be more efficient, the number of needed veterinarians will decrease substantially. On the other hand, if veterinarians are not careful, the probability of detection by surveillance visits will decrease, resulting in larger and longer lasting epidemics.

A peak for veterinarians was predicted very early in the epidemic (Figure 2). However, the assumption in the model is to be able to survey 450 farms a day in the protection and surveillance zones. If resources for this surveillance are reduced, as described by Halasa et al. (15), surveillance visits will be delayed, leading to delayed detections, prolonged epidemic duration, and an expected right shift in the peak for resources needed.

The resources estimated here were based on simulated epidemics and were shown to follow the simulated epidemic peaks closely (Figures 1, 2 and 5), however, with some delays for technicians and administrative personnel (Figures 3 and 4) and with an increase in needed resources again around day. Varying model inputs in the simulation model have previously been shown to change the outputs (5, 19) and corresponding changes in resources can be expected. Especially, the low risk contacts and the probability of local spread and disease detection were highly influential (19). Furthermore, a decrease in the length of the high-risk period (HRP) would decrease the size, duration, and costs of an outbreak (5). However, using a conservative estimate with a mode of 21 days (18–23) as the HRP would relate to the 2001 FMD epidemic in Europe, where the HRP was estimated to 21 days in the UK (26, 27) as well as in the Netherlands (28).

Our estimates were based on daily outputs from 981 simulated epidemics under a basic control strategy, e.g., the strategy expected to be used, if an outbreak would occur tomorrow. However, in very large epidemics, there is a probability that decision makers would not choose to stick to the basic control strategy, but would most likely add extra control measures such as preemptive culling or emergency vaccination, and therefore, the resources needed in the extreme epidemics would change.

Surprisingly, it was shown that there was a very high need for recruits from the DEMA used in the CD of detected herds (Figure 5), which might turn out to be a bottle neck; while our expectations were that the Danish Veterinary and Food Administration could run out of veterinarians.

Before the UK 2001 outbreak of FMD, the UK State Veterinary Service used two scenarios in their contingency planning, one moderate scenario with 10 simultaneous outbreaks and 1 severe outbreak, also with 10 simultaneous outbreaks, but with a large herd density. And they found a need for 235 veterinary officers, which they extrapolated to around 300 in more severe outbreaks. During the UK 2001 outbreak, 57 premises were infected before the first herd was diagnosed, leading to an almost immediate need for all state veterinary officers. Before the end of the outbreak, another 2,500 temporary veterinary inspectors were appointed, nearly 70 from abroad, and another 700 foreign government vets and secondees assisted in periods (29).

Based on the experience in the UK, we could fear that we are underestimating the needs during an FMD crisis in Denmark. However, even though we were interested in estimating manpower and materials needed, we were also aware that we can end up with even larger epidemics. Therefore, it was important for us to create a model, which can easily be adjusted during a crisis in an iterative process. Each time new information become available, regarding the epidemic or the resources needed, it can be fed into the model, and new outputs can be calculated. For example, if the compositions of the veterinary teams for different tasks are changed, we will change the inputs in the model and rerun it. Or if we rerun the stochastic model with historic data from the first 10 days of the epidemic, we will have more precise estimates on the further development of the epidemic and that can be put into the resource model.

The results of our estimations seem somewhat lower than what was estimated in Australia (17). Direct comparisons are difficult, due to geographical differences, resulting in several state disease control centers and local disease control centers in Australia, differences in estimated size of the epidemic, i.e., Garner et al. chose a 90th percentile epidemic and differences in how results are presented. Garner estimated nearly 20% of staff needed was veterinarians, while we estimated 33%.

The calculation of resources needed is an iterative process. The simulation model includes assumptions regarding resources, to simulate realistic epidemics, as scarce resources will prolong the epidemics. After assuming the available resources, we then calculate the daily needs. Naturally, this seems like a circular argument. However, in the simulation model, resources are roughly set as numbers of animals or herds that can be processed daily for either depopulation or surveillance. In the resource calculation presented here, we go into details regarding the teams for each task, the time needed, and look at number of herds and numbers of animals to process. The influence of the assumptions regarding resources has previously been described for depopulation (5) and surveillance (19). In both situations, the influence of reducing the resources was limited, reflecting that plenty of resources were assumed for most simulated outbreaks. This means that the calculations presented in this paper closely reflect the daily needs, when resources are not a limiting factor.

One of the assumptions was that all three veterinary regions would be involved from the beginning of the epidemic. While this was not truly realistic, the influence of this assumption was assumed to be limited, as many parameters even in the LCC depended on the numbers of herds and animals involved in the epidemic rather than the numbers of LCC. However, overall, we did estimate a clear decrease in the manpower needed for veterinarians as well as technicians and administrative staff. Therefore, an adjustment of the model taking region into account will be considered in future versions of the model.

In the current estimations, the very basic needs during an epidemic were estimated. Traveling time between herds was taken into account in the estimates (Table 1), while logistic challenges were not taken into account, such as veterinarians stuck in a herd after a surveillance visit that turned out to become a detection of an infected herd. In situations like that the veterinarian will stay in the detected herd and will not be able to visit other herds for the two following days. However, we assumed that the veterinarian would then be able to carry out other tasks, for example in the LCC. The competences needed for personnel involved in each task are described in details in the contingency plan for FMD (24) and in the project report (22). Furthermore, geographical challenges were not taken into consideration in these calculations. Denmark is a rather small country, where farmost destinations can be reached in a reasonable driving time (3–4 h). However, longer driving time will of course reduce the number of herds a veterinarian can visit on a given day. Nevertheless, estimating the amount of personnel needed gives us no answers in itself. To be able to use these estimates, it is necessary for the Danish Veterinary and Food Administration to compare with the present staff available and to consider how and where more personnel can be recruited to meet the needs during a crisis and which type of training is required in peace time, to be ready for an outbreak. The working group has continued working on this matter to update the Danish FMD contingency plan according to the results of the resource estimations and has given detailed descriptions on required competences for different types of staff for different tasks and how people can be trained to meet the challenges during a crisis. All of these results are described in a report from the expert group, in Danish (22).

## Author Contributions

AB, SM, and MJ in study design; AB and TH developed the model; and all the authors contributed to the results and discussion. AB wrote the manuscript, and all the authors commented and approved the final version.

## Conflict of Interest Statement

The authors declare that the research was conducted in the absence of any commercial or financial relationships that could be construed as a potential conflict of interest.

## Footnotes

^1^Of the 12 persons, 3 are included as authors, and the 9 other persons are listed in the acknowledgements.
