# Supplementary material for: Resource Estimations in Contingency Planning for Foot-and-Mouth Disease
Source: Front Vet Sci. 2017 May 11;4:64. doi: 10.3389/fvets.2017.00064 (PMC5425474; doi:10.3389/fvets.2017.00064)
Supplement: Supplementary file 1 [file Data_Sheet_1.PDF]

```
#### Input - resourcer ####

##First we define all inputs for each of the following


##Infected herds

##Traced herds

##Herds in protection zones

##Herds in surveillance zones

##Local crisis centers

##Danish Emergency Management Agency


#### Infected herds ####

  #### Taxation ####

  #One team = 3 taxators + 1 person from FVST (FVST=Veterinary and Food Administration)

                    VetPerTeamTax     <- 1     # Numbers of Vets in the team

                    VetTaxCattle      <- 1     # 1 team can taxate 1 herd per day (individual-taxation)

                    VetTaxSwine       <- 0.5   # 1 team can taxate 2 herds per day (group-taxation)

                    VetTaxSheep       <- 0.25  # 1 team can taxate 4 herds per day (group-taxation)

                    TechPerTeamTax    <- 3

                    TechTaxCattle     <- 1     # 1 team can taxate 1 herd per day (individual-taxation)

                    TechTaxSwine      <- 0.5   # 1 team can taxate 2 herds per day (group-taxation)

                    TechTaxSheep      <- 0.25  # 1 team can taxate 4 herds per day (group-taxation)

                    #Taxation of empty stables is not included

#### Culling ####

          VetCoordinating   <- 1      # 1 vet per herd

          Ownercontact      <- 1      # 1 technician per herd

          TimeCull          <- 8      # Effective daily working hours 8

                                      # (it is assumed that every culled farm will be

                                      #  finished the same day as culling is started)
```

```
NbCattlePerHour     <- 12          # 1 vet, 4 technicians, 1 truck driver for 12 cattle per hour
NbSheepPerHour      <- 20          # 1 vet + 1 technician, 20 sheep per hour
NbSowsPerHour       <- 30          # 30 sows per hour
NbFinishersPerHour  <- 60          # 60 finishers 30-120 kg per hour
NbWeanersPerHour    <- 300         # 300 weaners 0-30 kg. (assumed to be culled by use of CO2) per
                                   hour
NbWeanersPerSow     <- 5           # We estimate 5 weaners per sow
                                   # (not numbers of born but an average over all sows in the herd at
                                   #  a given time)
VetCullCattle       <- 1           # Each team can manage 12 cattle per hour
TechCullCattle      <- 5           # 4 technicians and 1 truck driver
VetCullSwine        <- 1           # Each team can manage 30 sows each hour
TechCullSwine       <- 10          # OR 60 finishers each hour
                                   # OR 300 weaners each hour
                                   # Tech = 2 technicians
                                   #     + 2 people to fixate animals
                                   #     + 4-6 people to drive the animals
                                   #     + 2 truck drivers
VetCullWeaners      <- 1
TechCullWeaners     <- 10          # Tech = 4 people to dump animals
                                   #     + 1 responsioble for managing gas
                                   #     + people to drive the animals
                                   #     + 1 truck driver
VetCullSheep        <- 1           # One team can manage 20 sheep per hour
TechCullSheep       <- 1
### Equipment - culling ####
SBoltCattle         <- 1           # Stock captive bolt pistol per team
MBoltCattle         <- 1           # Magnum captive bolt pistol per team
```

```
PatronCattle       <- 1.5           # Patrons per animal

SBoltSwine         <- 5             # Stock captive bolt pistol per team

MBoltSwine         <- 1             # Magnum captive bolt pistol per team

PatronSwine        <- 1.5           # Patrons per animal

ContainerCO2       <- 7000          # 1 container per 7000kg weaners, for anaesthesia

BoltSheep          <- 1             # Stock captive bolt pistol per team

PatronSheep        <- 1.5           # Patrons per animal

CleanPoint         <- 1                # 1 cleaning and disinfection point per detected cattle/swine herd
                                       in 48 hours

VetCleanPoint      <- 0.5           # 1 FVST-vet in 4 hours per cleaning and disinfection point

#### Equipment sampling ####

PCR             <- 60               # 60 PCR-samples per detected herd

Sero            <- 60               # 60 serological samples per detected herd

VetTest           <- 1              # Clinical insepction of all animals + taking samples

TechTest          <- 1              # Clinical insepction of all animals + taking samples


#### Rendering ####


ContainerCapacity     <- 15000  # 15T per container, 30T per transport

Containers            <- 500    # DAKA has 4-500 containers

                                # DAKA can render 30T per hour

WeightMalkeKo         <- 500    # Mean weight of a dairy cow2

WeightMalkeKalv       <- 100      # Mean weight of a calve in a dairy herd

WeightKødKo           <- 750    # Mean weight of a cow in a beef herd

WeightKødKalv         <- 150      # Mean weight of a calve in a beef herd

WeightSows            <- 250    # Mean weight of a sow

WeightFinisher        <- 70       # Mean weight of a finisher

                                   # (calculated from entrance in finisher section to slaughter)
```

```
WeightWeaners       <- 25      # Mean weight of a weaner
                               # (calculated from entrance in weaner section to movement out of
                                  weaner section)
WeightSheep         <- 65      # Mean weight of a sheep

#### Preliminary cleaning and disinfection ####
VetCleanCatSw       <- 1       # 1 person from FVST 1 full day per cattle/swine herd
VetCleanSheep       <- 0.25    # 1 person 2 hours in a sheep herd
DEMAClean           <- 1       # 1 team from DEMA - this is currently not used in the calculation,
                               #  just a notification that DEMA is also used in the cleaning!
#### Final cleaning and disinfection ####
VetCleanFinalCatSw  <- 5       # 1 FVST-vet in 5 days over a 21 day period per cattle/swine herd
VetCleanFinalSheep  <- 0.5     # 1 FVST-vet in ½ day over a 21 day period per sheep herd
DurationCleanFinal  <- 21
#### Suscpicions ####
SuspicionsPerDet    <- 5       # We assume 5 suspiscions per infected herd
                               # (these suspecions are non-infected)
VetSuspicion        <- 1       # 1 vet + 1 tehnician/adm 1 day in all suspicious herds
TechSuspicion       <- 1          # Currently, we are note using these values, as we do not know in
                                  which herd type these suspicions will occur
# VetSuspicionCatSw    <- 1    # 1 vet + 1 technician/adm 1 day per cattle/swine herd
# TechSuspicionCatSw   <- 1
# VetSuspicionSheep    <- 0.5  # 1 vet + 1 technician/adm ½ day per sheep herd
# TechSuspicionSheep   <- 0.5
#### Sureveyed herds ####
# Herds are visited as soon as ressourcer for sureveillance are ready,
# starting 1 day after they have been enroled in a zone
# assuming a capacity of 450 herds visited each day (PS + SZ + tracing surveillance)
```

```
# Herds in protection zones are visited twice, but this is included in the file - each herd occur as many times as visited

VetSurv <- 0.25 # clinical surveillance, 1 vet, 0.25 working day per herd

#### Local crisis center ####

LCC_Number <- 3 # We assumed that 3 LCCs were active during the full epidemic

# NOTE: THIS MIGHT BE CHANGED -> each LCC will be activated on first detection

# in an area, and will be active untill the end of the epidemic

LCC_VetLead <- 2 # THESE ARE PER LCC

LCC_LawLead <- 1 #

LCC_AdmLead <- 1 #

LCC_TechLead <- 1 #

LCC_HRLead <- 1 #

# THESE ARE RELATED TO NUMBERS OF ANIMALS/HERDS

LCC_TechPress <- 1 # 1 technician in each LCC

LCC_AdmLog <- 0.2 # Minimum 3, thereafter 1 per 5 herd

LCC_AdmCase <- 0.333 # Case-officers, 1 adm per 3 detected herds

LCC_VetSuspicionFirst <- 0.5 # 1 FVSTvet + ½ adm per 2 suspicions first 14 days

LCC_AdmSuspicionFirst <- 0.25

LCC_VetSuspicionAfter <- 0.333 # 1 FVSTvet + ½ adm per 3 suspicions first 14 days

LCC_AdmSuspicionAfter <- 0.167

LCC_AdmTaxating <- 0.5 # 1 adm per 2 detected herd

LCC_VetCull <- 0.5 # 1 vet per 2 detected herd

LCC_VetEPIFirst <- 0.333 # 1 FVSTvet + ½ adm per 3 suspicions first 14 days

LCC_AdmEPIFirst <- 0.167

LCC_VetEPIAfter <- 0.25 # 1 FVSTvet + ½ adm per 4 suspicions first 14 days

LCC_AdmEPIAfter <- 0.125

LCC_AdmScreening <- 0.02 # 1 adm per 50 herds

# THESE ARE per LCC:
```

```
        LCC_VetMoveDispFirst   <- 1      # 1 vet + 1 adm the first week

        LCC_AdmMoveDispFirst   <- 1      # 1 vet + 1 adm the first week

        LCC_VetMoveDispAfter   <- 8      # 8 vet + 24 adm after the first week

        LCC_AdmMoveDispAfter   <- 24     # 8 vet + 24 adm after the first week

        LCC_AdmService         <- 2      # 2 per 100 personnel

        LCC_TechIT             <- 2      # 2 tech per 100 personnel + 5 adm per 100 personnel

        LCC_AdmIT              <- 5      # 2 tech per 100 personnel + 5 adm per 100 personnel

        LCC_TechHR             <- 1      # personnel administration

        LCC_VetEdu             <- 1      # 1 vet per LCC

        LCC_AdmEdu             <- 1      # 1 adm per LCC

#### Danish Emergency Management Agency ####

        DaysPerHerd            <- 2      # Not used directly at the moment. Only for information.

        LeadOfficer            <- 2      # Leading officer, Management-1, Command-1

        Officers               <- 9      # Cleaning point-4(2-for persons, 2-for materiels), preliminary
                                         cleaning and dis.-3, Logistics-2

        Recruits               <- 47     # Command-3,Cleaning point-16(5-for persons, 11-for materiels),

                                         # preliminary cleaning and dis.-21, Logistics-4,Logistic support from
                                         BRC-3

#### Input from simulation model ####

setwd("C:/Users/anebo/Desktop/Ressources/")

  FarmFile      <- read.table("DataDADS.csv",header=T,sep=",")

  detected      <- read.table("Cat-DiagHerds.txt",header=TRUE,sep=" ")

  detected      <- detected[order(detected[,1],detected[,2]),]

  head(detected)

  detected      <- cbind(detected,

                FarmFile$herdCategory[detected[,3]],

                FarmFile$herdType[detected[,3]],

                FarmFile$sows[detected[,3]],

                FarmFile$finishers[detected[,3]],
```

```
                    FarmFile$herdSize[detected[,3]])
names(detected)    <-  c("iteration","day","HerdID","herdCategory","herdType","sows","finishers","herdSize")
depopulated       <- read.table("Cat-DiagHerds.txt",header=TRUE,sep=" ")
depopulated       <- depopulated[order(depopulated[,1],depopulated[,2]),]
depopulated       <-
cbind(depopulated[,1:3],FarmFile$herdCategory[depopulated[,3]],FarmFile$herdType[depopulated[,3]],
                    FarmFile$sows[depopulated[,3]],FarmFile$finishers[depopulated[,3]],
                    FarmFile$malCal[depopulated[,3]],FarmFile$bulls[depopulated[,3]],FarmFile$feCal[depopulated[,3]],
                    FarmFile$heif[depopulated[,3]],FarmFile$cows[depopulated[,3]],
                    FarmFile$herdSize[depopulated[,3]])
names(depopulated) <-  c("iteration","day","HerdID","herdCategory","herdType",
                   "sows","finishers",
                   "malCal","bulls","feCal",
                   "heif","cows",
                   "herdSize")

SurvHerds         <- read.table("Cat-SurvHerds.txt",header=F,sep=" ")
SurvHerds         <- SurvHerds[order(SurvHerds[,1],SurvHerds[,2]),]
SurvHerds         <- cbind(SurvHerds,
                    FarmFile$herdCategory[SurvHerds[,3]],
                    FarmFile$herdType[SurvHerds[,3]])
names(SurvHerds)   <-  c("iteration","day","HerdID","?","herdCategory","herdType")

Cat_FMD           <- read.table("Cat-FMD.txt",header=T,sep=" ")

#### Creating suspicion matrix ####
SuspicionMatrix   <- as.data.frame(matrix(0,ncol=1000,nrow=(395)))
## Find suspicions
```

```
for(j in 1:1000){
  Detect            <- detected[detected[,1]==j,,drop=FALSE]
  SuspicionDate      <- matrix(numeric(0),ncol=2)
  length(Detect[,1])
  sum(SuspicionDate[,2])


  for(i in 1:395){
    detect          <- Detect[Detect[,2]==i,,drop=FALSE]
     suspicions       <- length(detect[,3]) * SuspicionsPerDet
     if(suspicions>0){
        ## Finds a day of suspicion for each herd
       suspicionDate     <- ceiling(runif(ceiling(suspicions),min=i+1,max=i+10))
         SuspicionDate     <- rbind(SuspicionDate,cbind(suspicionDate,1))
         SuspicionDate2    <- SuspicionDate[order(SuspicionDate[,1]),]
          SuspicionDate3    <- aggregate(SuspicionDate2[,2],list(SuspicionDate2[,1]),FUN=sum)
          SuspicionMatrix[SuspicionDate3[,1],j] <- SuspicionDate3[,2]
      }
  }
  print(j)
}
## Saving the results as a file, so that we don't need to simulate suspicions every time,
## and we can reuse suspicions for sensitivity analyses
write.table(SuspicionMatrix, file="SuspicionMatrix.txt", col.names=F)
SuspicionMatrix <- read.table("SuspicionMatrix.txt", header=F, sep=" ")
## when I read in the matrix, I get the line numbers. So I remove the first column
SuspicionMatrix3 <- SuspicionMatrix[,-1]
names(SuspicionMatrix3) <- c(1:1000)
```

```
## I remove 19 iterations giving negative durations

negatives  <- Cat_FMD[which(Cat_FMD$gEpiDur<0),c(1,2)]

negatives2 <- c(as.numeric(row.names(negatives)),577)

SuspicionMatrix2  <- SuspicionMatrix3[,-negatives2]

SuspicionMatrix   <- SuspicionMatrix3


## Descriptives of other inputs

EpiStart        <- quantile(Cat_FMD$FirstEpiDet[Cat_FMD$gEpiDur>0],prob=c(0,0.05,0.25,0.5,0.75,0.95,1))

Duration        <- quantile(Cat_FMD$gEpiDur[Cat_FMD$gEpiDur>0],prob=c(0,0.05,0.25,0.5,0.75,0.95,1))

numDet          <- quantile(Cat_FMD$NumDet[Cat_FMD$gEpiDur>0],prob=c(0,0.05,0.25,0.5,0.75,0.95,1))

numCull         <- quantile(Cat_FMD$NumCulled[Cat_FMD$gEpiDur>0],prob=c(0,0.05,0.25,0.5,0.75,0.95,1))


## Numbers of suspicions for each iteration

numbSus <- colSums(SuspicionMatrix2)

numSusOut <- quantile(numbSus,prob=c(0,0.05,0.25,0.5,0.75,0.95,1))


## Removes the 18 "negative" scenarios from all files

detected    <- detected[!detected$iteration%in%negatives2,]

depopulated <- depopulated[!depopulated$iteration%in%negatives2,]


##*************************************************************************************************
****

## And now the fun starts :-)

#### Ressources ####

#### Defining outputs ####

Mat             <- matrix(numeric(0),ncol=23)

equipment        <- matrix(numeric(0),ncol=7)

#### Iteration loop ####
```

```
 for(j in unique(detected$iteration)){

  Detect <- detected[detected[,1]==j,,drop=FALSE]

  Depop  <- depopulated[depopulated[,1]==j,,drop=FALSE]

  SurvZ  <- SurvHerds[SurvHerds[,1]==j,,drop=FALSE]


  maxDay       <- max(Depop$day)

  firstDet     <- min(Detect$day)

  maxDetDay    <- max(Detect$day)

  newTime      <- numeric(0)


#### Time loop ####

    for(i in 1:395){


          detect     <- Detect[Detect[,2]==i,,drop=FALSE]

          depop      <- as.data.frame(Depop[Depop[,2]==i,,drop=FALSE])

          survZ      <- SurvZ[SurvZ[,4]==i,,drop=FALSE]

          depopPrev  <- Depop[Depop[,2]==(i-1),,drop=FALSE]

          newTime    <- ifelse(i<firstDet,0,i-(firstDet-1))


 vetCases      <- 0

 vetSuspicion  <- 0

 vetSurv       <- 0

 VetLCC        <- 0

 VetTotal      <- 0

 vetClean      <- 0

 vetCleanFinal <- 0

######################

#### Veterinarians ####
```

```
#######################

 #### VET cases ####

	## Vets to Taxation

	  VetCatTax    <- (sum(detect[,4]==1) * VetPerTeamTax * VetTaxCattle)

	  VetSwTax     <- (sum(detect[,4]==2) * VetPerTeamTax * VetTaxSwine)

	  VetShTax     <- (sum(detect[,4]==3) * VetPerTeamTax * VetTaxSheep)


	## Vets to Cull

	  VetCatCull   <- (sum(depop[,4]==1) * VetCoordinating) + ((sum(depop[depop[,4]==1,8]) / (TimeCull * 
NbCattlePerHour))*VetCullCattle)

	  VetSwCull    <- (sum(depop[,4]==2) * VetCoordinating) + ((sum(depop[depop[,4]==2,6]) / (TimeCull * 
NbSowsPerHour)) +

	               (sum(depop[depop[,4]==2,7]) / (TimeCull * NbFinishersPerHour)) +

	               ((sum(depop[depop[,4]==2,6]) * NbWeanersPerSow) / (TimeCull * 
NbWeanersPerHour))*VetCullSwine)

	  VetShCull    <- (sum(depop[,4]==3) * VetCoordinating) + ((sum(depop[depop[,4]==3,8]) / (TimeCull * 
NbSheepPerHour))*VetCullSheep)


	## Vets to CleanPoint

	  VetCleanP    <- (sum(depop[,4]%in%c(1,2)) * CleanPoint * VetCleanPoint)


	## Vets to Test

	  VetCatSwTest    <- (sum(depop[,4]%in%c(1,2)) * VetTest)


	## Vets to cleaning

	  VetCatSwClean   <- (sum(depop[,4]%in%c(1,2)) * VetCleanCatSw)

	  VetSheepClean   <- (sum(depop[,4]==3) * VetCleanSheep)


	  vetClean        <- ((sum(depop[,4]%in%c(1,2)) * VetCleanFinalCatSw)/DurationCleanFinal) +
```

```
                  ((sum(depop[,4]==3) * VetCleanFinalSheep)/DurationCleanFinal)
      vetCleanFinal   <- ifelse(vetClean>0,1,0)

                         ## Vets to cases, total

                         ## Remeber to add cleaning later!
  ## (because of the time related to cleaning over a 21-day period)
      vetCases  <- VetCatTax + VetSwTax + VetShTax +
            VetCatCull + VetSwCull + VetShCull +
            VetCleanP +
            VetCatSwTest +
            VetCatSwClean +
            VetSheepClean

 #### Vet Suspicions ####
      vetSuspicion      <- SuspicionMatrix[i,j] * VetSuspicion

#### Vet Surveillance ####
      vetSurv         <- length(survZ[,3]) * VetSurv

 #### Vet local crisis Center ####
      if((i > firstDet) & (i < (maxDay+30))){
       if(i < (firstDet + 14)){
        VetLCC        <- LCC_Number * (LCC_VetLead + LCC_VetEdu) +
                   (LCC_VetEPIFirst*length(detect[,3])) +
                   (LCC_VetCull*length(depop[,3])) +
                   (LCC_VetSuspicionFirst*SuspicionMatrix[i,j]) +
                    LCC_VetMoveDispFirst
```

```
            }
           if(i >= (firstDet + 14)){
            VetLCC        <- LCC_Number * (LCC_VetLead + LCC_VetEdu) +
                             (LCC_VetEPIAfter*length(detect[,3])) +
                             (LCC_VetCull*length(depop[,3])) +
                             (LCC_VetSuspicionAfter*SuspicionMatrix[i,j]) +
                              LCC_VetMoveDispAfter
            }
           }

        VetTotal     <- VetCatTax + VetSwTax + VetShTax +
                   VetCatCull + VetSwCull + VetShCull +
                   VetCleanP +
                   VetCatSwTest +
                   VetCatSwClean +
                   VetSheepClean +
                   vetSuspicion +
                   vetSurv +
                   VetLCC


#####################
#### Technicians ####
#####################

 techCases        <- 0
 techSuspicion    <- 0
 TechLCC          <- 0
```

```
TechTotal        <- 0

   ## Tech to Taxation
           TechCatTax  <- (sum(detect[,4]==1) * TechPerTeamTax * TechTaxCattle)
           TechSwTax   <- (sum(detect[,4]==2) * TechPerTeamTax * TechTaxSwine)
           TechShTax   <- (sum(detect[,4]==3) * TechPerTeamTax * TechTaxSheep)

   ## Tech to Cull
           TechCatCull <- (sum(depop[,4]==1) * Ownercontact) + ((sum(depop[depop[,4]==1,8]) / (TimeCull *
NbCattlePerHour))*TechCullCattle)
           TechSwCull  <- (sum(depop[,4]==2) * Ownercontact) + ((sum(depop[depop[,4]==2,6]) / (TimeCull *
NbSowsPerHour)) +
                       (sum(depop[depop[,4]==2,7]) / (TimeCull * NbFinishersPerHour)) +
                       ((sum(depop[depop[,4]==2,6]) * NbWeanersPerSow) / (TimeCull *
NbWeanersPerHour))*TechCullSwine)
           TechShCull  <- (sum(depop[,4]==3) * Ownercontact) + ((sum(depop[depop[,4]==3,8]) / (TimeCull *
NbSheepPerHour))*TechCullSheep)

   ## Tech to Test
           TechCatSwTest <- (sum(depop[,4]%in%c(1,2)) * TechTest)

#### Tech to cases, total ####
           techCases <- TechCatTax + TechSwTax + TechShTax +
                      TechCatCull + TechSwCull + TechShCull +
                      TechCatSwTest

 #### Tech Suspicions ####
           techSuspicion      <- SuspicionMatrix[i,j] * TechSuspicion

 #### Tech local krise Center ####
```

```
        if((i > firstDet) & (i < (maxDay+30))){
          TechLCC    <-  LCC_Number * (LCC_LawLead +
                               LCC_TechLead +
                               LCC_TechPress) +
                    LCC_TechHR
          }

          TechTotal  <-  TechCatTax+TechSwTax+TechShTax+
                    TechCatCull+TechSwCull+TechShCull+
                    TechCatSwTest+
                    techSuspicion +
                    TechLCC



########################
#### Administration ####
########################

  #### Adm krise Center ####
          AdmLCC   <- 0
          if((i > firstDet) & (i < (maxDay+30))){
           if(i < (firstDet + 14)){
            AdmLCC        <- LCC_Number * (LCC_AdmLead + LCC_HRLead + LCC_AdmEdu) +
                      ifelse((LCC_AdmLog*length(detect$CHR))<3,3,LCC_AdmLog*length(detect$CHR)) +
                      LCC_AdmCase*length(depop[,3]) +
                      LCC_AdmSuspicionFirst*SuspicionMatrix[i,j] +
                      LCC_AdmTaxating*length(depop[,3]) +
```

```
                   LCC_AdmEPIFirst*length(detect[,3]) +
                   LCC_AdmScreening * length(survZ[,3]) +
                   LCC_AdmMoveDispFirst
         }
        if(i >= (firstDet + 14)){
         AdmLCC       <-  LCC_Number * (LCC_AdmLead + LCC_HRLead + LCC_AdmEdu) +
                   ifelse((LCC_AdmLog*length(detect$CHR))<3,3,LCC_AdmLog*length(detect$CHR)) +
                   LCC_AdmCase*length(depop[,3]) +
                   LCC_AdmSuspicionAfter*SuspicionMatrix[i,j] +
                   LCC_AdmTaxating*length(depop[,3]) +
                   LCC_AdmEPIAfter*length(detect[,3]) +
                   LCC_AdmScreening * length(survZ[,3]) +
                   LCC_AdmMoveDispAfter
         }
        }

#### Updating Crisis center ####
      LCC_admService        <-  (VetTotal + VetLCC + TechTotal + AdmLCC)*LCC_AdmService/100
      LCC_techIT           <-  (VetTotal + VetLCC + TechTotal + AdmLCC)*LCC_TechIT/100
      LCC_admIT            <-  (VetTotal + VetLCC + TechTotal + AdmLCC)*LCC_AdmIT/100
      TechTotal2           <-  TechTotal + LCC_techIT
      AdmLCC2              <-  AdmLCC + LCC_admService + LCC_admIT
##############
#### DEMA ####
##############
      DEMATotal      <- numeric(0)
  ## DEMA for cleaning
      DEMALeadOfficer  <- (length(depop[,3])+length(depopPrev[,3])) * LeadOfficer
```

```
            DEMAOfficers     <- (length(depop[,3])+length(depopPrev[,3])) * Officers

            DEMARecruits     <- (length(depop[,3])+length(depopPrev[,3])) * Recruits

                                                  DEMATotal       <- DEMALeadOfficer + DEMAOfficers + DEMARecruits

##############

#### DAKA ####

##############

                    TotWeight      <- 0

                    containers     <- 0

                    WeightRuminants <- 0

                    WeightSwine     <- 0


        if(dim(depop)[1]>0){

          depop$KGWeight   <- rep(0,dim(depop)[1])

          IndexM <- depop[,5]==1

          Temp1  <- ((depop[IndexM,10] + depop[IndexM,12] + depop[IndexM,13]) * WeightMalkeKo) + 
((depop[IndexM,11]+depop[IndexM,9])*WeightMalkeKalv)

          if(length(Temp1)>0) depop$KGWeight[IndexM] <- Temp1

          IndexK <- depop[,5]==2

          Temp2 <- ((depop[IndexK,10] + depop[IndexK,12] + depop[IndexK,13]) * WeightKødKo) + 
((depop[IndexK,11]+depop[IndexK,9])*WeightKødKalv)

          if(length(Temp2)>0) depop$KGWeight[IndexK] <- Temp2

          IndexG <- depop[,4]==2

          Temp3 <- (depop[IndexG,6] * WeightSows) + (depop[IndexG,7]*WeightFinisher) +(depop[IndexG,6] * 
WeightWeaners * NbWeanersPerSow )

           if(length(Temp3)>0) depop$KGWeight[IndexG] <- Temp3

          IndexF <- depop[,4]==3

          Temp4 <- (depop[IndexF,8] * WeightSheep)

          if(length(Temp4)>0) depop$KGWeight[IndexF] <- Temp4

          depop$Containers <-  depop$KGWeight / ContainerCapacity
```

```
TotWeight      <- sum(depop$KGWeight)
WeightRuminants <- sum(depop$KGWeight[depop[,4]%in%c(1,3)])
WeightSwine    <- sum(depop$KGWeight[depop[,4]==2])
containers     <- sum(ceiling(TotWeight / ContainerCapacity))
}
```

```
#### EQUIPMENT ####
          sBolt       <- 0
          mBolt       <- 0
          Patrons     <- 0
          cleanPoints  <- 0
          containerCO2  <- 0
          Index <- depop[depop[,1]==j & depop[,2]==i,3]
          if((i < maxDay) & (i >= firstDet)){
           sBolt      <- sum(depop[,4]==1) * SBoltCattle +
                     sum(depop[,4]==2) * SBoltSwine +
                     sum(depop[,4]==3) * BoltSheep
           mBolt      <- sum(depop[,4]==1) * MBoltCattle +
                     sum(depop[,4]==2) * MBoltSwine
           Patrons     <- sum(depop[depop[,4]==1,13]) * PatronCattle +
                     sum(depop[depop[,4]==2,13]) * PatronSwine +
                     sum(depop[depop[,4]==3,13]) * PatronSheep
           containerCO2 <- ceiling(sum(depop[depop[,4]==2,6])*NbWeanersPerSow * WeightWeaners /
ContainerCO2)
           cleanPoints  <- (length(depop[,3])+length(depopPrev[,3])) * CleanPoint
          }
          equipment <- rbind(equipment,cbind(j,i,sBolt,mBolt,Patrons,containerCO2,cleanPoints))
```

```
## Preparing outputs
  Mat <- rbind(Mat,cbind(j,i,newTime,
                    vetCases,vetSuspicion,vetSurv,VetLCC,VetTotal,
                                                                          vetClean,vetCleanFinal,

techCases,techSuspicion,TechLCC,TechTotal2,
                                                                          AdmLCC2,

DEMALeadOfficer,DEMAOfficers,DEMARecruits,DEMATotal,

TotWeight,WeightRuminants,WeightSwine,containers))
 }
  ##Making it possible to follow how many iterations have been run
  print(j)
}

#### Saving outputs in Data ####
Data <- as.data.frame(Mat)
head(Data)
## Creating new variables to be used for updating with inclusion of vets for final cleaning and desinfection
## This is done seperately, as this task is estimated to take 5 working days in total,
## but distributed over 21 days.

Data$vetCleanFinal2     <- Data$vetCleanFinal
Data$vetCases2          <- Data$vetCases
Data$VetTotal2          <- Data$VetTotal
Data$TechLCC2           <- Data$TechLCC
Data$TechTotal3         <- Data$TechTotal2
Data$AdmLCC3            <- Data$AdmLCC2
```

```
## Distributing the calculated ressource for final cleaning and desinfection over 21 days

for(a in 1:dim(Data)[1]){

  if(Data$vetCleanFinal[a]==1){

    Data$vetCleanFinal2[(a+1):(a+20)] <- Data$vetCleanFinal2[(a+1):(a+20)] + Data$vetCleanFinal[a]

   }

 }


## Updating ressources after the cleaning was updated in the previous step

for(a in 1:dim(Data)[1]){

  Data$vetCases2[a]          <- Data$vetCases[a] + Data$vetCleanFinal2[a]

  Data$VetTotal2[a]          <- Data$VetTotal[a] + Data$vetCleanFinal2[a]

  Data$TechLCC2[a]           <- Data$TechLCC2[a] + Data$vetCleanFinal2[a]*LCC_TechIT/100

  Data$TechTotal3[a]         <- Data$TechTotal3[a] + Data$vetCleanFinal2[a]*LCC_TechIT/100

  Data$AdmLCC3[a]            <- Data$AdmLCC3[a] + Data$vetCleanFinal2[a]*(LCC_AdmService+LCC_AdmIT)/100

}

write.table(Data, file="SkaleringsOutputDTU-DADS.txt", sep=" ", col.names=T)
```
